# The High Testosterone Concentrations of the Bucks Used in the “Male Effect” Is Not a Prerequisite for Obtaining High Ovarian Activity in Goats from Mediterranean Latitudes

**DOI:** 10.3390/ani12080954

**Published:** 2022-04-07

**Authors:** Luis Ángel Zarazaga, María-Carolina Gatica, Ignacio De La Rosa, Manuel Delgado-Pertíñez, José Luis Guzmán

**Affiliations:** 1 Departamento de Ciencias Agroforestales, Escuela Técnica Superior de Ingeniería, “Campus de Excelencia Internacional Agroalimentario, CeiA3”, Campus Universitario de la Rábida, Universidad de Huelva, Carretera de Huelva-Palos de la Frontera, s/n, 21819 Huelva, Spain; ignacio.delarosa@dcaf.uhu.es (I.D.L.R.); guzman@uhu.es (J.L.G.); 2 Facultad de Recursos Naturales Renovables, Universidad Arturo Prat, Avenida Arturo Prat, Iquique 2120, Chile; mgatica@unap.cl; 3 Departamento de Agronomía, Escuela Técnica Superior de Ingeniería Agronómica, Universidad de Sevilla, Ctra. Utrera km 1, 41013 Sevilla, Spain; pertinez@us.es

**Keywords:** male effect, testosterone, photoperiod, GnRH agonist, odor, scrotal circumference

## Abstract

**Simple Summary:**

The introduction of males to anovulatory females previously separated from males (or even without separation) can induce estrous activity within a few days; this is termed the “male effect”. It is a common reproductive management practice on the extensive and semi-extensive goat farms of the Mediterranean area. High testosterone concentrations at the moment of the induction of the “male effect” are usually considered essential to obtain a high reproductive response from female goats. By controlling the secretion of GnRH, it is possible to control the gonadotropins and testosterone secretion. GnRH agonists have been used to control testosterone concentration in different species. Two experiments were carried out to determine if the contact during the seasonal anestrous of the males with the females modifies the testosterone concentrations and if the use of a GnRH agonist on the bucks to avoid variation in testosterone concentrations when the male effect is induced causes variation in the reproductive response of the does.

**Abstract:**

Two experiments were carried out. Firstly, 54 anestrous females were placed in contact with photostimulated males (Photo; *n* = 27) or with no photostimulated males (Natural; *n* = 27). Moreover, a group of bucks treated with artificial photoperiod and a group of bucks subjected to natural photoperiod without contact with females was used (Photo Isolated and Natural Isolated, respectively). In the Natural groups, the testosterone concentrations were similar except for three days after the introduction of the bucks to the does (19.72 ± 4.11 vs. 2.05 ± 0.25 ng/mL for Natural and Natural isolated bucks, respectively, *p* < 0.05). However, no differences were observed in the Photo groups during the entire experiment. The percentage of females showing estrous was higher in the group of females in contact with photostimulated bucks (96 vs. 74%, respectively, *p* < 0.05). In the second experiment, a GnRH agonist, deslorelin, was used to regulate the testosterone concentrations of the bucks. Seventy anestrous females were divided into five groups depending on the treatment received by the bucks to which they were exposed: photostimulated bucks (Photo group, *n* = 14); photostimulated bucks but treated with the agonist at the onset of the photoperiod treatment (Photo-Ago Long group, *n* = 13); photostimulated bucks but treated with the agonist at the end of the photoperiod treatment (Photo-Ago Short group, *n* = 15); bucks receiving no photostimulation but treated with the agonist at the end of the photoperiod treatment period (Natural-Ago Short group, *n* = 13) and bucks receiving no photostimulation nor agonist (Natural group, *n* = 15). The agonist treatment increased testosterone concentrations after the injection, which remained high for the entire experiment (*p* < 0.05). Six days after the introduction of the bucks to the does, the testosterone concentrations increased only in the Natural group reaching similar concentrations to the other groups (12.17 ± 6.55, 16.69 ± 4.53, 8.70 ± 0.61, 11.03 ± 1.45 and 14.42 ± 3.64 ng/mL for Photo, Photo-Ago Long, Photo-Ago Short, Natural-Ago Short and Natural bucks, respectively, *p* > 0.05). No differences in reproductive parameters were observed (*p* > 0.05). These results demonstrate that, at Mediterranean latitudes, anestrous females can stimulate the testosterone concentrations of bucks after a period of isolation. The high testosterone concentrations are not a prerequisite for an adequate response to the male effect.

## 1. Introduction

Reproductive activity in Mediterranean goats shows a clear annual pattern, with a period of breeding activity that begins in the late summer or autumn and a period of seasonal anestrous that starts in late winter or at the onset of spring [1,2,3]. The timing of reproductive seasonality in these small ruminants, as well as in most temperate zone mammals, is controlled by the photoperiod [4]. This relationship leads to variation in the availability of their products (milk and meat) over the year. However, reproduction outside of the breeding season would allow milk and meat to be produced throughout the year, in accordance with commercial requirements and consumer expectations.

To overcome this seasonality, one of the most used techniques is the manipulation of socio-sexual relationships. The introduction of males to anovulatory females previously separated from males (or even without separation) [5] can induce estrous activity within a few days; this is termed the “male effect”. The male effect is an effective management practice for inducing ovulation in seasonally anovulatory goats. Since it is very easy to set up and low cost, the male effect is widely used in extensive and semi-extensive goat production systems in Mediterranean countries. The female response to the male effect depends on many factors; the sexual behavior of the males is one of the most important [6].

In bucks in temperate zones, sexual behavior is expressed seasonally during autumn and winter. The onset of the behavior is preceded by approximately 6 weeks by the increase in testosterone levels from 2 to 20 ng/mL [7,8]. To ensure bucks are sexually active can easily be simulated using long day photoperiods [6,9,10,11,12,13,14]. In bucks, this artificial lighting regime generates increased levels of testosterone secretion, which in turn influences the production of pheromones [15]. Following photostimulation, complete sexual activity in bucks is observed, with full libido, increased scrotal circumference, and higher semen quality [16,17]. This increased sexual behavior could explain the improved reproductive results when photostimulated bucks are used to induce the male effect. However, our group has observed a very high ovarian activity in Mediterranean goats when non-photostimulated bucks are used. In those bucks, increased testosterone concentrations were observed following their introduction to the does [18].

Other management techniques can stimulate testosterone concentrations. These techniques have been used to observe that exposure to estrous females resulted in rapid increases in LH secretion in rams [19,20] and goat bucks [21,22], but anestrous ewes elicited a reduced response [23,24,25]. This endocrine response depends upon the seasonal period, and the response is maximized during the non-breeding season (in rams: [20,26]; in bucks: [21]) and reduced during the breeding season [27].

In relation to the secretory patterns of reproductive hormones in the male. A pulse of GnRH released from the hypothalamus releases a pulse of LH from the pituitary, which in turn elicits a burst of testosterone secretion from the testis. The increased testosterone then suppresses GnRH and LH, completing an atypical negative feedback loop [28]. By controlling the secretion of GnRH, it is possible to control the gonadotropins and testosterone secretions, and thus, fertility [29,30]. GnRH agonists have been used to control testosterone concentration in dogs and bulls [31,32]. Slow-release implants of the synthetic GnRH agonist, deslorelin, have been developed to suppress fertility and sexual behavior in dogs, where these implants reliably inhibit gonadotropin release and spermatogenesis for several months [33,34]. A complete loss of reproductive function, including spermatogenesis, has also been reported in boars treated with slow-release deslorelin implants [35,36].

Slow-release deslorelin treatment induces increased LH and testosterone secretion during a short period of some hours or days in various species (rams: [37,38]; dogs: [39]; stallions: [40]). However, the sustained application of the agonists induces a decrease in the pituitary content of LH and FSH [41] and, therefore, testosterone secretion [37,38]. Deslorelin is the most widely used GnRH agonist [34,42,43]. However, the effects of deslorelin in small ruminants have been examined in only a few studies of rams [37,38] and bucks [43]; thus, there is no information on how controlling testosterone in male goats affects fertility in female goats.

We hypothesized that for goats at Mediterranean latitudes, the high testosterone concentrations of the bucks used to induce the male effect are not essential to obtain a high ovarian response in the does. The objectives of this work were to examine (i) if the contact during the seasonal anestrous of the males with the females modifies the testosterone concentrations (ii) if the high testosterone concentrations of the bucks at introduction are essential to induce a high ovarian activity response in the does, and (iii) if using a GnRH agonist (deslorelin) on the bucks to avoid variation in testosterone concentrations when the male effect is induced causes variation in the reproductive response of the does, and whether the fertility of the does bred with deslorelin-treated bucks is modified.

## 2. Materials and Methods

### 2.1. Study Conditions

This study was carried out at the experimental farm of Universidad de Huelva (Spain) (latitude 37°20’ N and longitude 6°54’ W), which meets the requirements of the European Community Commission for Scientific Procedure Establishments (2010/63). The animals were kept—under intensive animal husbandry—in closed housing with windows to the outside and free access to an open area. Any procedure on the animals was done by qualified staff and following the national guidelines covered by the basic applicable regulations for the protection of animals used for experimentation and other scientific purposes (Royal Decree 53/2013 of 1 February 2013). The Ethics Committee on Animal Research (CEEA) of Universidad de Granada (297-CEA-OH-2018) supervised all study experiments, authorized by the Directorate-General for Agriculture and Livestock Production of the Andalusian regional government (Reference number 22/05/2019/094).

### 2.2. Experiment 1

The objective of this experiment was to demonstrate the first described objective (i): that contact with the females stimulates an increase in testosterone concentrations in males.

#### 2.2.1. Treatment of Bucks

This study used two groups (*n* = 7 each) of sexually experienced bucks of Blanca Andaluza and Murciano-Granadina breeds, which were randomly distributed between the groups and were 2.23 ± 0.17 years old at the beginning of the study. On 19 November, a group of these males, housed in open barns, was exposed to a long photoperiod (16 h light, 8 h dark; lights on 6:00, lights off 22:00) for 84 days (Photo group). Fluorescent white light tubes connected to an electronic timer that delivered at least 200 lux at eye level, supplied the hours of additional artificial light needed to provide artificial long-day photoperiod to male goats. At the end of the photoperiod treatment (February 11 of the following year), the animals were kept under natural photoperiod. Bucks of the other group were subjected to natural photoperiod (Natural group) throughout the experimental period. Both groups of bucks were kept in separate corrals.

#### 2.2.2. Experimental Design

On 22 March, 39 days after the end of the photoperiod treatment, eight bucks (four from each group of bucks) were introduced to 54 Blanca Andaluza seasonally anovulatory and non-pregnant goats (2–3 years old at the beginning of the study) and were used until 25 April (for 34 days) according to the following treatments: four bucks of the Photo group were introduced to 27 females (Photo Used group), and four bucks from the Natural group were put in contact with 27 females (Natural Used group). Bucks were equipped with marking harnesses. The other bucks (3 per group) were completely isolated from females and each other and formed the Photo-Isolated and Natural-Isolated groups, depending on the photoperiod treatment.

### 2.3. Experiment 2

The objective of this experiment was to demonstrate the second described objective (ii) if the high testosterone concentrations of the bucks at introduction are essential to induce a high ovarian activity response in the does, and (iii) if using a GnRH agonist (deslorelin) on the bucks to avoid variation in testosterone concentrations when the male effect is induced causes variation in the reproductive response of the does, and whether the fertility of the does bred with deslorelin-treated bucks is modified.

#### 2.3.1. Preparation of Bucks

Five groups (*n* = 2 each) of sexually experienced bucks of Blanca Andaluza and Murciano-Granadina breeds, which were evenly distributed between groups, were used to induce the male effect and were 2.81 ± 0.11 years old at the beginning of the study. On 21 November, six males were housed in open barns under an artificial photoperiod as described in Experiment 1. At the end of the photoperiod treatment (i.e., on 13 February of the following year), these bucks were maintained under natural photoperiod conditions. Within the photoperiod-treated bucks, one group (*n* = 2) received the photoperiod treatment alone (Photo group), and two groups (four bucks, two per group) received one subcutaneous implant containing 7.4 mg of deslorelin (Suprelorin; Virbac, Barcelona, Spain), a continuously released GnRH agonist (Ago). A single subcutaneous implant was inserted in the pre-scapular zone of each buck and remained in situ throughout the study. One of these groups (*n* = 2) received the implant on the first day of photoperiod treatment (Photo-Ago Long group). Towards the end of the photoperiod treatment, on 30 January, the other group (*n* = 2) received the subcutaneous implant (Photo-Ago Short group). The other two groups (*n* = 2 each) of bucks that did not receive photoperiod treatment were exposed to the natural photoperiod throughout the experiment; one group received one subcutaneous implant of Suprelorin on 30 January (Natural-Ago Short group), and the other did not receive any treatment (Natural group). The bucks in each group were housed in separate pens.

#### 2.3.2. Experimental Design

On 27 March, 43 days after the end of the photoperiod treatment, bucks were introduced to 70 seasonally anovulatory Blanca Andaluza does, which were 2–3 years old at the beginning of the study, until 24 April (for 28 days). The males introduced to the females were equipped with marking harnesses and placed with the experimental females. Five subgroups were formed, according to the treatment of the males:

(1) 14 does exposed to the bucks of the Photo group.
(2) 13 does exposed to the bucks of the Photo-Ago Long group.
(3) 15 does exposed to the bucks of the Photo-Ago Short group.
(4) 13 does exposed to the bucks of the Natural-Ago Short group.
(5) 15 does exposed to the bucks of the Natural group.

#### 2.3.3. Buck Odor and Scrotal Circumference

The smell of buck was determined once a week (always by the same person) following the technique described by Walkden-Brown et al. [44]. Odor was assessed at a distance of 10–15 cm from the posterior base of the horns and categorized as follows: 0 (neutral odor or not different from that of a doe or a castrated buck), 1 (light odor), 2 (moderate odor), or 3 (intense odor). Scrotal circumference was measured once a week by passing a tape measure (±0.1 cm) around the scrotum at the point of maximum diameter of the testicles.

### 2.4. Methodology Common to Both Experiments

#### 2.4.1. Management

Once a day, all animals received barley straw (ad libitum), alfalfa hay, and a commercially available concentrate to cover their maintenance nutritional needs according to the Institut National de la Recherche Agronomique (INRA) and considering a live weight of 50 and 75 kg for does and bucks, respectively [45]. The animals had unrestricted access to water and vitamin-mineral blocks.

#### 2.4.2. Detection of Estrous Behavior and Ovulation

Once bucks (wearing a harness) were introduced, signs of estrus were observed daily in does [46]. The time between the introduction of bucks and the first heat was also calculated for each doe.

Blood samples from the jugular vein were collected in tubes with 10 µL of heparin. Immediately after, samples were centrifuged at 2300× *g* for 30 min and the plasma was stored at −20 °C for further analysis of progesterone plasma levels.

For three consecutive weeks (Day 0; March 22 and March 27 for Experiments 1 and 2, respectively) and before introducing the bucks, blood samples were obtained from does to determine their estrous cycle stage. Progesterone levels ≤1.0 ng/mL in all samples would indicate the doe was anestrus. Neither doe was excluded from the study after the assessment of progesterone concentrations. Does were randomly assigned to the different experimental groups.

To monitor fertility response after the introduction of the bucks, blood samples were collected from does every 48–72 h during the first 10 days, and twice a week after that. A normal corpus luteum was established for progesterone plasma concentrations ≥1.0 ng/mL for at least two consecutive samples [47]. Occurrence of ovulation was established as the day progesterone concentration was ≥1.0 ng/mL for the first time. Silent ovulation was defined as progesterone levels ≥1.0 ng/mL in at least one sample without heat. We calculated the percentages of does showing signs of heat, with or without ovulation, and silent ovulations based on the profiles of progesterone plasma concentrations.

#### 2.4.3. Analysis of Plasma Progesterone

For progesterone plasma concentration determination an ELISA assay kit (Ridgeway Science Ltd., Gloucester, UK) was used following the manufacturer’s instructions [48]. The sensitivity of the assay was 0.2 ng/mL. Intra-assay coefficients of variations for 0.5 ng/mL and 1 ng/mL sample pools were 5.3% and 5.9% and 6.5% and 7.0%, for Experiment 1 and 2, respectively. Inter-assay coefficients of variations for 0.5 ng/mL and 1 ng/mL sample pools were 9.3% and 7.8% and 7.9% and 7.3%, for Experiment 1 and 2, respectively.

#### 2.4.4. Fecundity, Fertility, Prolificacy, and Productivity

Pregnancy was determined by transrectal ultrasound scanning 45 days after the detection of heat [49]. The following was calculated: fecundity (pregnant does × 100/does with heat and ovulation); fertility (does that gave birth × 100/total number of does in each experimental group), prolificacy (born kids/does that gave birth); and productivity (number of kids born in each experimental group/does in each experimental group).

#### 2.4.5. Buck Plasma Testosterone

Testosterone levels in bucks were determined from plasma collected following the method previously described for measuring progesterone in does. Blood samples were taken weekly from bucks in both experiments, before being put together with the does; after their introduction, blood samples were taken twice a week. For testosterone plasma concentration determination an ELISA assay kit (Demeditec Diagnostics, Kiel-Wellsee, Germany) was used. The sensitivity of the assay was 0.1 ng/mL. Intra-assay coefficients of variations for 0.2 ng/mL and 6.0 ng/mL sample pools were 6.9% and 9.3% and 2.6% and 2.0%, for Experiments 1 and 2, respectively. Inter-assay coefficients of variations for a pool of samples of 0.2 ng/mL and 6.0 ng/mL were 6.4% and 6.0% and 2.0% and 3.6%, for Experiments 1 and 2, respectively.

#### 2.4.6. Sexual Behavior of the Bucks

The sexual behavior of the bucks was also observed for 30 min (from 8:00 to 8:30) on the first 10 days (Days 0–9) following the placement of the bucks with the does (Day 0 = the day of introduction). Genital sniffs (when the buck sniffed the anogenital area of the doe); licks (when the buck licked the flanks of the doe); nudges (when the buck kicked the doe); sneezing sounds (bucks emitting a sneezing sound); mounting attempts (when the buck attempted to mount the doe without intromission) and mounts (when the bucks mounted the doe with intromission) were all recorded. The sexual behavior of all bucks was monitored using a video recording system, thus minimizing human interaction with the animals.

### 2.5. Statistical Analyses

Data are presented as means ± standard error. The values for testosterone were examined using an ANOVA with time as a repeated measure and the experimental groups as the main factors. For each experiment, the model included fixed between-subjects experimental factors and a fixed within-subject factor for time (repeated measures), as well as the interactions between these factors. The linear model used for Experiment 1 was as follows:

Yijkl = μ + Pi + Rj + (P × R)ij + Tk + (T × P)ik + (T × R)jk + (T × P × R)ijk + εijkl(1)

where Yijkl is the value of the dependent variable; μ is the overall mean; Pi is the fixed between-subjects effect of treatment of the bucks (i = Photo, *n* = 7 or Natural, *n* = 7); Rj is the fixed between-subjects effect if the bucks were used or not [j = Used, *n* = 8 (Photo, *n* = 4 or Natural, *n* = 4) or Isolated, *n* = 6 (Photo, *n* = 3 or Natural, *n* = 3)]; Tk is the within-subject fixed effect of time (28 records from each buck); P × R, T × P, T × R, T × P × R are the interactions among these factors; and εijkl is the residual error.

For the testosterone concentrations, odors and scrotal circumferences in Experiment 2, the linear model used was as follows:

Yijkl = μ + Pi + Rj + (P × R)ij + Tk + (T × P)ik + (T × R)jk + (T × P × R)ijk + εijkl(2)

where Yijkl is the value of the dependent variable; μ is the overall mean; Pi is the fixed between-subjects effect of treatment of the bucks (i = Photo, *n* = 6 or Natural, *n* = 4); Rj is the fixed between-subjects effect if the bucks were treated with the agonist [j = agonist, Ago Long, *n* = 2 or Ago Short, *n* = 4 (Photo, *n* = 2 or Natural, *n* = 2), without agonist, *n* = 4 (Photo, *n* = 2 or Natural, *n* =2)]; Tk is the within-subject fixed effect of time (33 samples for testosterone, 25 records for scrotal circumference and odor); P × R, T × P, T × R, T × P × R are the interactions among these factors; and εijkl is the residual error. In both experiments, the Duncan test was used to detect. weekly differences.

The variables expressed as percentages—females ovulating, females expressing estrous behavior and ovulating, fecundity, and fertility—were analyzed using the Fisher–Freeman–Halton exact probability test for multiple group comparisons and the Fisher exact probability test for two-group comparisons as required. Odor and prolificacy were compared using the Kruskal–Wallis test for global comparisons between all experimental groups.

Productivity and the number of days between male introduction and ovulation, or ovulation with estrous behavior, were compared using ANOVA with the treatment of the bucks for each experiment as fixed effects. The linear model used for each parameter was as follows:

Yij = μ + Pi + εij(3)

where Yij is the value of the dependent variable; μ is the overall mean; Pi is the fixed between-subjects effect of treatment of the bucks (i = Exp 1: Photo, *n* = 27 or Natural, *n* = 27; Exp 2: Photo, *n* = 14, Photo-Ago Long, *n* = 13, Photo-Ago Short, *n* = 15, Natural-Ago Short, *n* = 13, Natural, *n* = 15), and εij is the residual error.

The Pearson or Spearman correlation coefficient was calculated for the weekly testosterone concentrations, odors, and scrotal circumferences as required. For correlations, the significance was set at *p* ≤ 0.01.

The percentage of genital sniffs, licks, nudges, sneezing sounds, mounting attempts, and mounting with intromission were calculated for each group and analyzed using the Fisher exact probability test for two-group comparisons. All calculations were performed using SPSS Statistics for Windows (version 25.0; IBM Corp., Armonk, NY, USA). The significance was set at *p* ≤ 0.05.

## 3. Results

### 3.1. Experiment 1

#### 3.1.1. Testosterone Concentrations and Sexual Behavior

**Figure 1:**
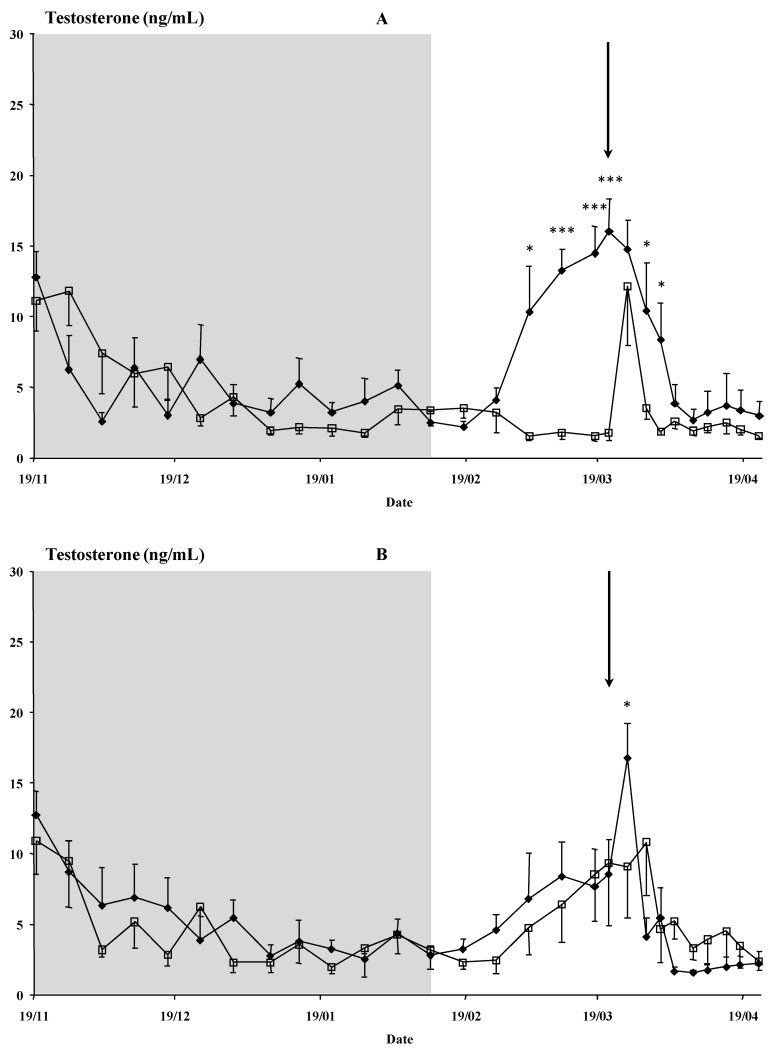
(Experiment 1). (**A**) Plasma testosterone concentrations (ng/mL) of males submitted to a photoperiod treatment, i.e., long days for three months between November and February (◆, *n* = 7) and untreated males (☐, *n* = 7). (**B**) Plasma testosterone concentrations (ng/mL) of males (both photoperiod-treated, i.e., long days for three months between November and February, and untreated males) used (◆, *n* = 8) or not used (☐, *n* = 6) to induce the male effect. The shaded area indicates the photoperiod treatment time. The arrow indicates the moment of the introduction of the bucks. * *p* < 0.05. *** *p* < 0.001 in the same day differ significantly between male groups.

Time had a marked effect on the plasma testosterone concentration (*p* < 0.001), as did the interaction of time × buck photoperiod treatment (*p* < 0.001) (Figure 1A). A clear effect of the buck treatment (Photo vs. Natural) was observed (*p* < 0.05) (Figure 1A). The bucks of the Photo groups had greater testosterone concentrations than the bucks of the Natural groups from 4 March until 1 April (at least *p* < 0.05) except for 25 March (Figure 1A). An effect due to the interaction of time × buck used or not used to induce the male effect (*p* < 0.05) was observed (Figure 1B). In general, the testosterone concentration of both groups, used or not, was similar for the entire experiment, except when the bucks were introduced to the females (25 March), at which time the testosterone concentrations of the bucks used increased and decreased rapidly (Figure 1B).

**Figure 2:**
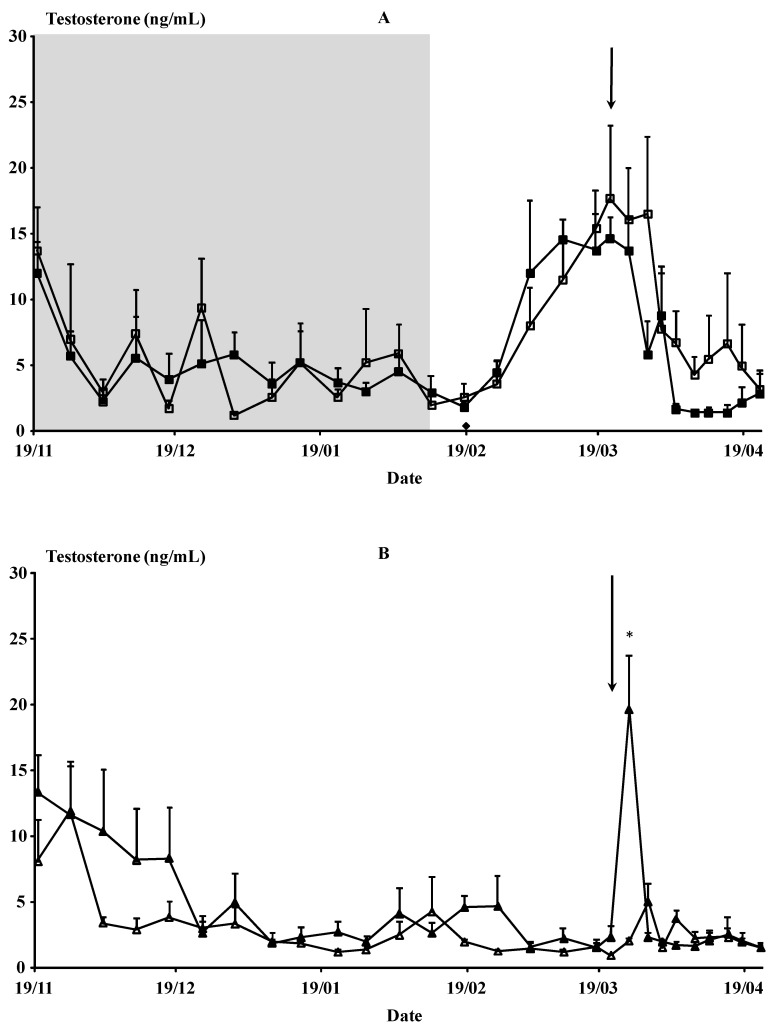
(Experiment 1). (**A**) Plasma testosterone concentrations (ng/mL) of (**A**) males submitted to photoperiod treatment, i.e., long days for 3 months between November and February (◼, *n* = 4; ☐, *n* = 3) and (**B**) untreated males (▲, *n* = 4; △, *n* = 3). Filled symbols indicate the males used to induce the male effect, and empty symbols indicate those not used and isolated from any contact with females. The shaded area indicates the photoperiod treatment period. The arrow indicates the moment of the introduction of the bucks. * *p* < 0.05 in the same day differ significantly between male groups.

No differences at any point were observed between the photoperiod-treated bucks (Photo Used vs. Photo Isolated) (*p* > 0.05) (Figure 2A). Similar results were found for the groups under natural photoperiod (Natural Used vs. Natural Isolated), except on 25 March, three days after the introduction of the bucks to the does for induction of the male effect (*p* < 0.05) (Figure 2B).

**Figure 3:**
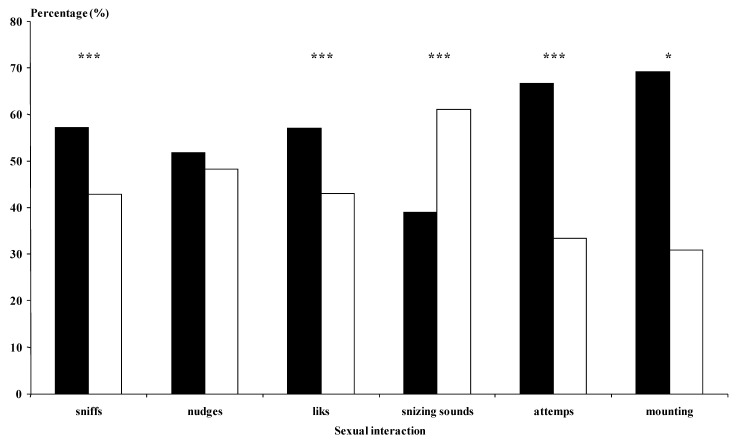
(Experiment 1). Types of sexual advances (%) experienced by females submitted to the male effect using photoperiod-treated males, i.e., long days for three months between November and February (◼, *n* = 7) or males treated with natural photoperiod (☐, *n* = 7). * *p* < 0.05. *** *p* < 0.001 in the same sexual advance differ significantly between male groups.Sniffs (when the buck sniffed the anogenital area of the doe). Nudges (when the buck kicked the doe); Licks (when the buck licked the flanks of the doe); Sneezing sounds (sounds emitted by the bucks); Mounting attempts (when the buck attempted to mount the doe without intromission); Mounts (when the bucks mounted the doe with intromission).

The bucks of the Photo group performed more genital sniffs, licking, mounting attempts, and mounts with intromission than the Natural bucks (at least *p* < 0.05; Figure 3).

#### 3.1.2. Female Reproductive Response

The treatment of bucks modified significantly the percentage of does showing estrous (*p* < 0.05), which was higher in the group of does submitted to the male effect by photostimulated bucks (Table 1). No other differences between groups were observed in the reproductive parameters studied (Table 1).

### 3.2. Experiment 2

#### 3.2.1. Testosterone Concentrations, Odor, Scrotal Circumference, and Sexual Interactions with Bucks

**Figure 4:**
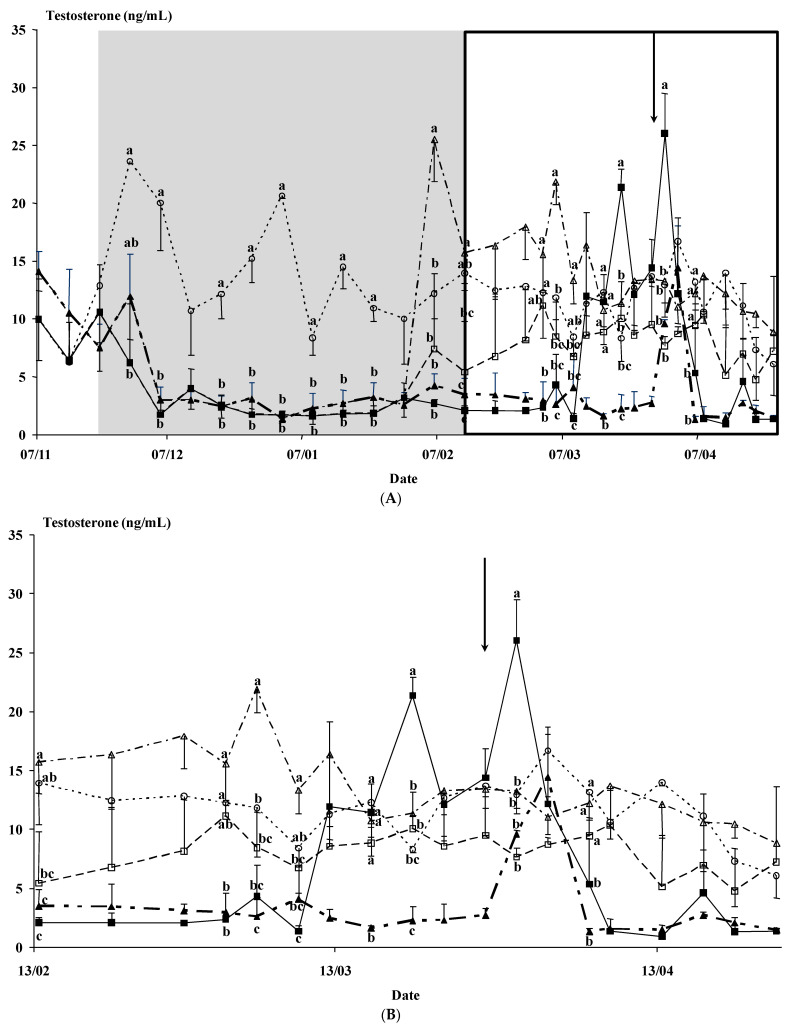
(**A**). (Experiment 2). Plasma testosterone concentrations (ng/mL) of males were used to induce the male effect. The groups were: males submitted to a photoperiod treatment, i.e., long days for three months between November and February (◼, *n* = 2, Photo); males submitted to the same photoperiod treatment and given a GnRH agonist at the onset of the photoperiod treatment (🞅, *n* = 2, Photo-Ago Long); males submitted to the same photoperiod treatment and given a GnRH agonist at the end of the photoperiod treatment (**☐**, *n* = 2, Photo-Ago Short); males subjected to the natural photoperiod and a GnRH agonist at the same time as the Photo-Ago Short (**△**, *n* = 2, Natural-Ago Short) and males subjected to the natural photoperiod (▲, *n* = 2, Natural). The shaded area indicates the photoperiod treatment duration. The inset figure in the box indicates that it will be magnified for better viewing (**B**). The arrow indicates the moment of the introduction of the bucks. Different letters in the same day differ significantly between male groups, *p* < 0.05. (**B**) (Experiment 2). This figure is a magnification of the box period presented in (**A**), between the end of the photoperiod treatment and the end of the experiment. The description of the groups is the same as in (**A**). Plasma testosterone concentrations (ng/mL) of males were used to induce the male effect. The groups were: males submitted to a photoperiod treatment, i.e., long days for three months between November and February (◼, *n* = 2, Photo); males submitted to the same photoperiod treatment and given a GnRH agonist at the onset of the photoperiod treatment (🞅, *n* = 2, Photo-Ago Long); males submitted to the same photoperiod treatment and given a GnRH agonist at the end of the photoperiod treatment (**☐**, *n* = 2, Photo-Ago Short); males subjected to the natural photoperiod and a GnRH agonist at the same time as the Photo-Ago Short (**△**, *n* = 2, Natural-Ago Short) and males subjected to the natural photoperiod (▲, *n* = 2, Natural). The arrow indicates the moment of the introduction of the bucks. Different letters in the same day differ significantly between male groups, *p* < 0.05.

Time had a marked effect on the plasma testosterone concentration (*p* < 0.01), as did the interaction of time × buck photoperiod treatment (*p* < 0.01), the interaction of time × agonist (*p* < 0.01), and the interaction of time × buck photoperiod treatment × agonist (*p* < 0.01) (Figure 4A,B). Moreover, a clear effect of the use of an agonist (*p* < 0.05) and the interaction of buck photoperiod treatment × agonist (*p* < 0.05) was observed. The interactions observed were due to the insertion of the agonist implant (three Ago groups), inducing a rise in testosterone concentrations at the time of insertion. These testosterone concentrations were high during the entire experiment. Moreover, the concentrations of the Natural group of bucks were low except for a rapid increase immediately following their introduction to the does; then, the concentrations were similar to the Photo group of bucks.

When the testosterone concentrations during the week before the buck introduction and the week after the buck introduction (Figure 4A,B) were compared, no differences within each group were observed except in the Natural group, which had higher testosterone concentrations after the introduction of the bucks (2.41 ± 0.52 vs. 8.45 ± 2.59 ng/mL for testosterone concentrations before the male effect and after the male effect, respectively, *p* < 0.05).

**Figure 5:**
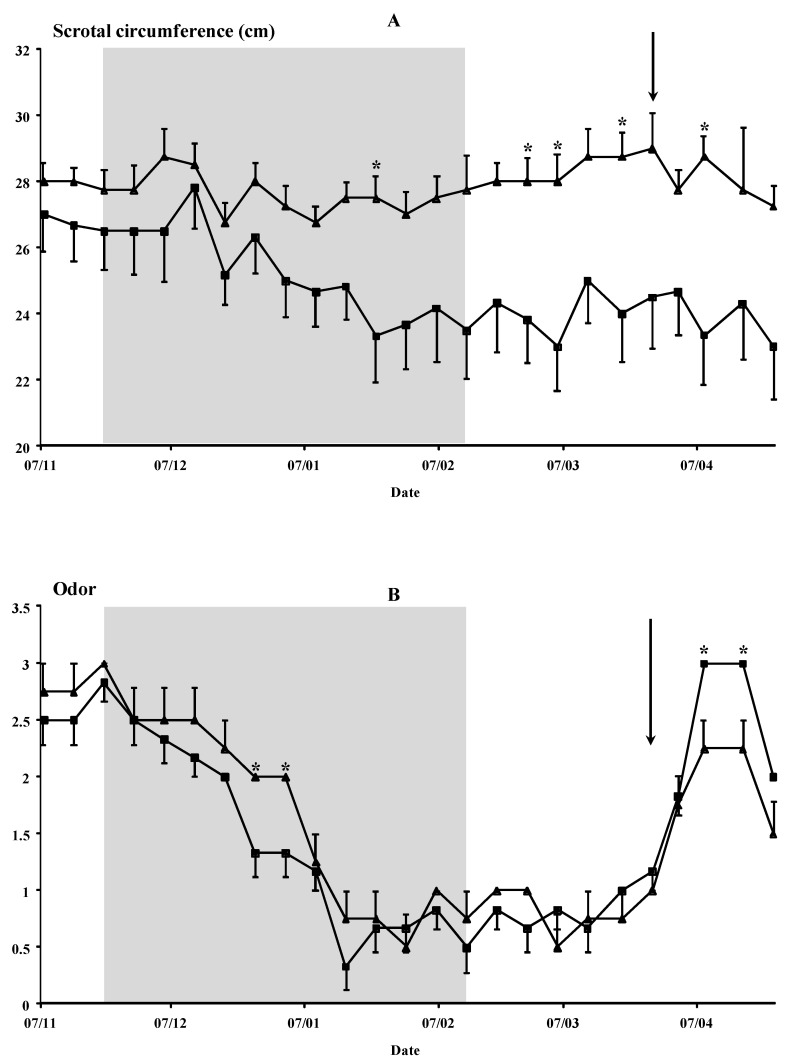
(Experiment 2). (**A**) Scrotal circumferences and (**B**) odors of the males are used to induce the male effect. These males were submitted to a photoperiodic treatment, i.e., long days for three months between November and February (◼, *n* = 6, Photo groups) or to the natural photoperiod (▲, *n* = 4, Natural groups). The shaded area indicates the photoperiod treatment duration. The arrow indicates the moment of the introduction of the bucks. * *p* < 0.05 in the same day differ significantly between male groups. Buck smell was evaluated subjectively according to the scale described by Walkden-Brown et al. [44], allocating a score of 0 (neutral odor, no different from a female or a castrated male), 1 (mild male odor), 2 (moderate male odor) or 3 (strong male odor).

Time had a significant effect on odor and scrotal circumference (*p* < 0.01), as did the interaction of time × buck photoperiod treatment for the odor parameter (*p* < 0.05) and scrotal circumference (*p* < 0.01) (Figure 5). The odor was stronger in the Natural bucks during the photoperiod treatment (26 December and 2 January) than in the photoperiod-treated bucks. However, it was higher in the Photo bucks at the end of the experiments (8 and 17 April) than in the Natural bucks. The scrotal circumference was higher in the Natural bucks than in the photoperiod-treated bucks, especially at the end of the photoperiodical treatment. No other interaction was observed between the time × agonist or time × buck photoperiod treatment × agonist (*p* > 0.05). Similarly, no effect of the buck treatment, agonist, or interaction between both sources of variation was observed (*p* > 0.05).

The odor correlated positively to the testosterone concentrations in the Natural group (r = 0.563, *p* < 0.001). The scrotal circumference correlated negatively in the Photo-Ago Short group (r = −0.834, *p* < 0.001).

**Figure 6:**
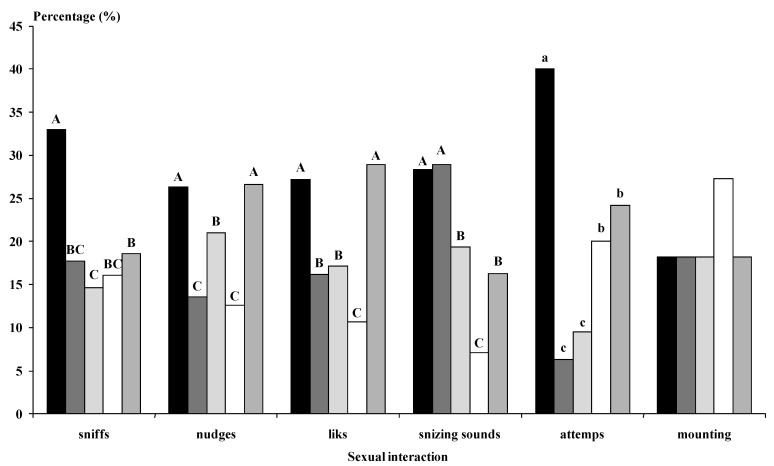
(Experiment 2). Types of sexual advances (%) experienced by females submitted to the male effect using photoperiod-treated males, i.e., long days for three months between November and February (Photo, ◼); males submitted to the same photoperiod treatment and a GnRH agonist at the onset of the photoperiod treatment (Photo-Ago Long, ◼); males submitted to the same photoperiod treatment and a GnRH agonist at the end of the photoperiod treatment (Photo-Ago Short, ◼); males subjected to the natural photoperiod (Natural, ☐) and males subjected to the natural photoperiod and a GnRH agonist at the same time as the Photo-Ago Short (Natural-Ago Short, ◼). Different letters in the same sexual advance differ significantly. Lower case (a, b, c) indicates differences *p* < 0.05 in the whole groups and capital letters (A, B, C) indicate differences *p* < 0.01 in the whole groups. Sniffs (when the buck sniffed the anogenital area of the doe). Nudges (when the buck kicked the doe)*;* Licks (when the buck licked the flanks of the doe)*;* Sneezing sounds (sounds emitted by the bucks)*;* Mounting attempts (when the buck attempted to mount the doe without intromission)*;* Mounts (when the bucks mounted the doe with intromission).

The bucks of the Photo group performed more genital sniffs, nudges, licking, sneezing sounds (*p* < 0.01), and mounting attempts (*p* < 0.05) than the Natural group bucks (Figure 6). In general, the photoperiod-treated bucks implanted with the agonist (Photo-Ago Long and Photo-Ago Short) performed fewer sexual interactions than the Photo bucks. However, the Natural-Ago Short bucks experienced more sexual interactions in the form of nudges, licks, and sneezing sounds than the Natural group (*p* < 0.01) (Figure 6).

#### 3.2.2. Female Reproductive Response

No differences between groups were observed for the reproductive parameters studied (Table 2).

## 4. Discussion

The results obtained confirm our expectations. In Experiment 1, the results demonstrate that contact with females induces a rapid rise in the testosterone concentrations of the bucks exposed to a natural photoperiod; however, this contact did not modify the testosterone concentrations of the photostimulated bucks. This result was confirmed in Experiment 2. In the second experiment, the agonist used induced a rapid and sustained increase in testosterone concentrations determined from the time of implant placement, which avoided any modification of testosterone concentrations when the males were in contact with the females. In this second experiment, the testosterone increased only in the bucks from the Natural group. The agonist did not affect fertility.

The experiments were performed for two consecutive years. As detailed in the material and methods, the management system and nutrition were the same and the climatic conditions were very similar between years. Concerning the possible effect of age on the bucks, in both experiments, the bucks had very similar ages. Ungerfeld et al. [50] observed that adult rams (4–6 years old) induce a greater ovulation percentage and estrous response in ewes, resulting in greater ovulation numbers, and pregnancy and conception rates in comparison to yearling rams. It could be supposed that the number of males in the second experiment was very low, only 2 bucks per group. However, the SEM of the testosterone concentrations in the figures was low and similar between groups and to groups of experiment 1, suggesting that the response was homogenous in all groups of experiment 2. Secondly, we tried to have a similar ratio of males:females between the experimental groups in the first (1:6.75) and second experiment (between 1:6.5 and 1:7.5). Nevertheless, those ratios are higher than the ratios used for livestock but according to previous results did not influence the reproductive results [12].

In both experiments, the testosterone concentrations of the bucks under natural photoperiod increased three days after the introduction of the bucks to the does. However, the mean onset of estrous in these experiments was around 3 to 6 days after buck exposure. As a consequence, contact with anestrous females was able to stimulate the testosterone concentrations. The exposure to estrous females resulted in rapid increases in LH secretion in rams [19,20] and bucks [21,22]. But, anestrous ewes triggered a reduced response [23,24]. However, in the present experiment and others performed by our group [18], we observed a similar response, suggesting that at Mediterranean latitudes or with the Spanish breeds used, the anestrous does can induce a rapid increase in testosterone concentration, observed just after buck introduction, more quickly than the estrous response of the females. These results suggest that the bucks isolated from the does are stimulated by them even when they are anestrous. Moreover, this effect could have masked a higher seasonality of buck testosterone concentrations, in experiments where estrous females were used to obtain ejaculates from bucks [11,51].

Concerning the three groups treated with the GnRH agonist, different reasons could justify its use in this study. The Photo-Ago Long group allows the determination of whether the physiological perception of a stimulating photoperiod is modified by the testosterone concentration. The Photo-Ago Short group received a long photoperiod without any modification of testosterone concentrations during the photoperiod treatment. The third group, Natural-Ago Short, was submitted the natural photoperiod but underwent physiological modification two months before the male effect was applied. In all cases, the use of the agonist prevented any modification of the testosterone concentrations when the bucks were in contact with the females, probably due to overexposure of the GnRH system to the agonist. As a consequence, the objective of this use in the experimental design was appropriate. In these groups, the testosterone concentrations when the male effect was performed were high and similar to the Photo group at the moment of the buck introduction, even in the bucks submitted to the natural photoperiod.

The use of the GnRH agonist induced a rapid rise in testosterone concentrations. This effect was observed even during the photoperiod treatment that usually induces a decrease in testosterone concentrations. This effect was also observed when the GnRH agonist was implanted at the end of the photoperiod treatment or in bucks subjected to a natural photoperiod when the testosterone concentrations are typically low. The most interesting effect of this agonist was the prevention of increased testosterone concentrations when the bucks were in contact with the females in three different groups of bucks.

Giriboni et al. [43] recently used this agonist and observed a similar effect on testosterone concentrations, which were higher three and four months following implant insertion. However, there are some differences between the work of Giriboni et al. [43] and the present work. First, the former was performed over a long period, seventeen months, and to compare those results with the results of our present experiment, only the first five months. Second, for the previous work, this period included the breeding season; however, in our present work, it was the onset of the seasonal anestrous, and this was enhanced by a long day photoperiod treatment. Third, the sampling frequency was much higher in our present study, which allows a more accurate description of the effect of the agonist on testosterone concentrations.

In Experiment 1 only the percentage of females showing estrous was higher in the female group in contact with photostimulated males. In Experiment 2, the Natural group showed similar results to the other groups, with different, higher testosterone concentrations when the males were introduced to the females. This result differed from other results obtained by our group under similar conditions [13,14]. The main reason for this result could be due to the use of Murciano-Granadina bucks. This breed has reduced seasonality. We have demonstrated that the photoperiod treatment is more effective when was applied to bucks of a breed with an intense seasonal anestrous, such as the Blanca Andaluza breed [18]. This fact could demonstrate that under Mediterranean conditions, the testosterone concentrations of the bucks when they are introduced to the does is not a primary factor in obtaining an adequate reproductive response.

Fecundity or fertility was not modified by the treatment of the bucks with the agonist. This result could indicate that this GnRH agonist does not induce a reduction in the semen quality and agrees with the results of Giriboni et al. [43], who did not observe complete azoospermia.

In both our experiments, the sexual interactions between the bucks and the does were more frequent in the group of does using photostimulated bucks. However, the more intensive sexual interactions did not yield a higher reproductive response in the females. Moreover, in the second experiment, the agonist treatment in the Photo groups did not induce a higher number of sexual interactions than the Photo group without agonist treatment. Even the Photo group showed higher numbers of sexual interactions (sniffs, licks, nudges, and mounting attempts) than any of the groups treated with the agonist. These results suggest that elevated testosterone concentration is not required to induce more sexual behavior. All the agonist-treated bucks showed high testosterone concentrations; however, they did not perform more interactions than the Photo group. Perhaps the treatment of photoperiod stimulated some mechanism that the GnRH agonist could overcome due to the overstimulation of the testosterone secretion.

Odor correlated positively with the testosterone concentrations in the Natural group; however, in the groups treated with the agonist, no correlation was observed. Although the agonist treatment modified the testosterone concentrations, this modification did not induce changes in the odor of the bucks. It has been suggested that increases in buck odor are associated with sustained elevations in mean testosterone concentrations of over 2 ng/mL [52]. This action of testosterone appears to be mediated by increases in sebaceous gland activity [44,53]. It is possible that this effect was not induced in the present experiment due to the agonist treatment. In the present experiment, the correlations of the scrotal circumference with odor or testosterone concentration are not very consistent. Similarly, Walkden-Brown et al. [44] did not observe any such correlations in well-nourished bucks.

## 5. Conclusions

The results of the present study demonstrate that, at Mediterranean latitudes, the contact of the anestrous does with the bucks following a period of isolation induces a rapid but short increase in testosterone concentrations. The ovarian response of the does in contact with this kind of buck is similar to that of does in contact with photostimulated bucks with high testosterone concentrations at their introduction. Consequently, a high testosterone concentration is not a prerequisite to obtaining a reproductive response when the females are submitted to the male effect. The agonist deslorelin was used to regulate the testosterone concentrations and did not reduce fertility when used in bucks to induce the male effect.

## Figures and Tables

**Table 1:** (Experiment 1). Reproductive responses of females submitted to the male effect using photoperiod-treated males, i.e., long days for three months between November and February (Photo) or males subjected to natural photoperiod (Natural).

Variable	Natural (*n* = 27)	Photo (*n* = 27)	Significance ^1^
Females ovulating (%)	89	100	NS
Interval between the introduction of males and normal ovulation	10.21 ± 0.75	11.04 ± 0.58	NS
Females in estrous and ovulating (%)	74	96	*
Interval between the introduction of males and estrous	6.50 ± 0.58	6.36 ± 0.48	NS
Ovulation rate	1.57 ± 0.13	1.78 ± 0.14	NS
Fecundity (%)	85	61	NS
Fertility (%)	56	52	NS
Prolificacy	1.53 ± 0.13	1.50 ± 0.14	NS
Productivity	0.85 ± 0.17	0.78 ± 0.16	NS

Females ovulating (percentage of does with at least one progesterone concentration measurement above the baseline of 1.0 ng/mL); Interval between the introduction of males and normal ovulation (interval between buck placement with does and the first progesterone concentration above the baseline of 1.0 ng/mL); Females in estrous and ovulating (percentage of does showing estrous with at least one progesterone concentration above the baseline of 1.0 ng/mL); The interval between the introduction of males and estrous (interval between the time of buck placement with does and the first detected estrous); Ovulation rate (number of corpora lutea observed in each doe by transrectal ultrasonography conducted 6 to 8 days after the detection of estrous). Fecundity (percentage of pregnant does per does showing estrous and ovulating); Fertility (percentage of does kidding per does in each mating group); Prolificacy (the number of kids born per female kidding); Productivity (the number of kids born per doe in each mating group). ^1^ Significance: NS, Non-significant; *, *p* < 0.05).

**Table 2:** (Experiment 2). The reproductive response of the females submitted to the male effect using photoperiod-treated males, i.e., long days for three months between November and February (Photo); males submitted to the same photoperiod treatment and a GnRH agonist at the onset of the photoperiod treatment (Photo-Ago Long); males submitted to the same photoperiod treatment and a GnRH agonist at the end of the photoperiod treatment (Photo-Ago Short); males subjected to the natural photoperiod and a GnRH agonist at the same time as the Photo-Ago Short (Natural-Ago Short) and males subjected to the natural photoperiod (Natural).

Variable	Photo (*n* = 14)	Photo-Ago Long (*n* = 13)	Photo-Ago Short (*n* = 15)	Natural-Ago Short (*n* = 13)	Natural (*n* = 15)
Females ovulating (%)	100	92	100	92	100
Interval between the introduction of males and normal ovulation (days)	6.36 ± 0.46	5.83 ± 0.50	6.07 ± 0.34	6.58 ± 0.66	5.20 ± 0.36
Females in estrous and ovulating (%)	93	85	93	85	100
Interval between the introduction of male and estrous (days)	4.62 ± 0.72	3.45 ± 0.80	3.07 ± 0.54	3.27 ± 0.66	2.67 ± 0.40
Fecundity (%)	92	64	93	73	93
Fertility (%)	86	54	87	62	80
Prolificacy	1.75 ± 0.18	1.86 ± 0.14	1.31 ± 0.13	1.63 ± 0.18	1.50 ± 0.15
Productivity	1.50 ± 0.23	1.00 ± 0.28	1.13 ± 0.17	1.00 ± 0.25	1.20 ± 0.20

Females ovulating (percentage of does with at least one progesterone concentration above the baseline of 1.0 ng/mL); The interval between the introduction of males and normal ovulation (interval, days, between buck placement with does and the first progesterone concentrations above the baseline of 1.0 ng/mL); Females in estrous and ovulating (percentage of does showing estrous with at least one progesterone concentration above the baseline of 1.0 ng/mL); The interval between the introduction of males and estrous (interval, days, between the time of buck placement with does and the first detected estrous); Fecundity (percentage of pregnant does per does showing estrous and ovulating); Fertility (percentage of does kidding per does in each mating group); Prolificacy (the number of kids born per female kidding); Productivity (the number of kids born per doe in each mating group).

## Data Availability

The data presented in this study are available on request from the corresponding author.
